# Evaluation of the modified 3Mix‐Simvastatin combination in non‐instrumental endodontic therapy of necrotic primary molars: A two‐arm randomized controlled trial

**DOI:** 10.1002/cre2.860

**Published:** 2024-03-03

**Authors:** Walaa Almarji, Mohannad Laflouf, Yasser Alsayed Tolibah

**Affiliations:** ^1^ Department of Pediatric Dentistry, Faculty of Dentistry Damascus University Damascus Syria

**Keywords:** dental pulp necrosis, lesion sterilization and tissue repair, pulpectomy, root canal therapy

## Abstract

**Objective:**

This study aimed to assess the clinical and radiographic outcomes of non‐instrumentation endodontic treatment (NIET) using a modified antibiotic mix of cefixime, ciprofloxacin and metronidazole with simvastatin (an anti‐inflammatory, bone regeneration drug) on necrotic primary molars compared to conventional pulpectomy to help preservation of necrotic primary teeth until its natural exfoliation.

**Materials and Methods:**

Forty mandibular primary second molars with necrotic pulp tissue from 38 healthy patients aged between 4 and 8 years were randomly assigned to two groups with a 1:1 allocation ratio. Group A teeth underwent conventional root canal treatment. The procedure involved a two‐visit approach, employing k‐files and h‐files during the initial visit, followed by the application of calcium hydroxide paste as canal dressing between visits, while Group B teeth were treated with 3Mixtatin. All teeth were clinically evaluated after 1, 3, 6, and 12 months, and radiographically at 3, 6, and 12 months. Two external examiners assessed the results. Data analysis was conducted using a chi‐square test at a 0.05 significance level.

**Results:**

At the end of the follow‐up interval, 90% of teeth in each group exhibited no clinical signs or symptoms. Additionally, inter‐radicular radiolucency healing occurred in 75% of cases in the NIET group and 89.5% in the conventional pulpectomy group. However, no statistically significant difference was found between the two groups.

**Conclusion:**

NIET using 3Mixtatin seems to be a good alternative choice to conventional pulpectomy, offering a less complex treatment approach that may help avoid the complications associated with traditional pulpectomy and could be suitable for teeth with shorter roots.

## 1 INTRODUCTION

The optimal preservation of primary teeth is widely acknowledged as the most effective means of ensuring proper space maintenance for their successors (Pinky et al., 2011). Furthermore, untreated carious lesions have been demonstrated to elevate the likelihood of developing new cavities in both primary and permanent dentition, ultimately leading to premature tooth loss (Fuks & Nuni, 2019). This issue also correlates with an increased frequency of emergency room visits and school absences, consequently impacting the oral health‐related quality of life (Coll et al., 2020).

Primary teeth with infected root canals often present significant clinical challenges (Moura et al., 2021). The factors represent in the complex root canal morphology with accessory and secondary canals in primary molars, the behavioral issues in children, the unattainable complete chemo‐mechanical removal of necrotic pulp tissue and obturation the primary molars root canal system (Shetty et al., 2020).

The alterations in the root tip position due to the gradual resorption of the roots of primary molars by the succedaneous tooth bud or periapical tissues inflammation pose a substantial challenge for both general practitioners and pediatric dentists when conducting conventional root canal therapy for primary molars with necrotic pulp (Arangannal et al., 2019; May, 2016; Moskovitz & Tickotsky, 2016). Additionally, the existing materials have yet to fulfill all of the suitable root canal system obturation materials criteria for primary teeth (Agarwal et al., 2019; Shetty et al., 2020).

A novel technique called lesion sterilization and tissue repair (LSTR) or non‐instrumentation endodontic treatment (NIET) has been proposed as a biologically less complicated alternative to pulpectomy for treating necrotic primary molars (Rajsheker et al., 2018). The development of this concept is credited to the Cariology Research Unit at Niigata University School of Dentistry in Japan, which relies entirely on chemical sterilization without the use of instrumental or mechanical disinfection (May, 2016). It involves the application of a combination of antibiotics to eradicate bacteria from dental caries, non‐vital pulps, and carious dentin in the root of primary teeth (Takushige et al., 2004). Nevertheless, the inclusion of minocycline in this mixture may result in discoloration, particularly in calcifying teeth, representing a potential drawback (Burrus et al., 2014).

LSTR aims to eliminate or reduce the microbial load within the infected pulp, preventing further damage and infection. Additionally, LSTR facilitates tissue repair by promoting the healing and regeneration of the damaged pulp tissue to restore the integrity and functionality of the affected tooth (Moreira et al., 2022).

The clinical effectiveness of LSTR or NIET techniques has been substantiated with notable success (Nanda et al., 2014; Takushige et al., 2004). Furthermore, statins, recognized for their cholesterol‐lowering properties, demonstrate regenerative potential in dentistry (N. A. Aminabadi et al., 2016; Naser Asl Aminabadi et al., 2013; Jamali et al., 2018). Notably, research indicates that statins can augment osteoblast and odontoblast function, while also exerting anti‐inflammatory effects conducive to bone regeneration (Chak et al., 2022). Consequently, the integration of statins into the triple antibiotic paste emerges as a promising strategy for enhancing inter‐radicular lesion healing.

After reviewing the published literature, it was observed that there is a diversity in the disinfectants, antibiotics used, and the methods of applying the NIET technique, which led to variations in the clinical and radiographic outcomes of this technique (Anila et al., 2020; Burrus et al., 2014; Coll et al., 2020). This identified variability has served as the impetus for undertaking this study, aiming to assess the efficacy of LSTR employing the modified 3Mix with simvastatin in necrotic primary molars.

## 2 MATERIALS AND METHODS

### 2.1 Study design, settings, and ethical approval

From January 2018 to March 2021, this study was conducted at the Department of Pediatric Dentistry at the Faculty of Dentistry, Damascus University, Damascus, Syria. The study was a single‐center interventional double‐blinded randomized clinical trial that employed a two‐arm parallel superiority design with a 1:1 allocation ratio. The study adhered to the ethical guidelines outlined in the Declaration of Helsinki and obtained ethical approval from the Local Research Ethics Committee of the Faculty of Dentistry (Approval No. UDDS‐394‐08072018/SRC‐1450). The project was self‐funded and registered at the ISRCTN registry under ID number 12940165. This RCT was written according to Preferred Reporting Items for Randomized Trials in Endodontics (PRIRATE) 2020 guidelines (Nagendrababu et al., 2020).

### 2.2 Recruitment and eligibility criteria

#### 2.2.1 Participant's inclusion criteria

The study included only those children between the ages of 4 and 8 who had previously exhibited cooperative or definitely cooperative behavior based on Frankel's Behavioral Rating Scale, during their dental treatment.

#### 2.2.2 Participant's exclusion criteria

These include cases of facial cellulitis, non‐cooperative patients, those with a history of allergy to one of the drugs being used, and individuals with systemic disease or under antibiotic regimen for the past 2 weeks.

#### 2.2.3 Teeth inclusion criteria

Primary lower second molars with necrotic pulp and evidence of pulp vitality loss: buccal swelling (gingival abscesses) or fistula openings, pathological mobility, spontaneous pain or tenderness to percussion, bifurcation radiolucency with minimum 2 mm of the intact healthy bone surrounding the permanent tooth bud, pathological root resorption.

#### 2.2.4 Teeth exclusion criteria

These include non‐restorable teeth, excessive root resorption involving more than half of the root on radiographs, perforation of the pulpal floor, and teeth exhibiting pre‐shedding mobility.

#### 2.2.5 Clinical examination

All the primary molars that met the inclusion criteria were assessed clinically before the treatment and during all follow‐up intervals by two experienced pediatric dentists (M.L. and Y.A.T), any disagreement between them was resolved with help from a third experienced examiner. All signs and symptoms were recorded before the treatment and during all follow‐up intervals.

#### 2.2.6 Radiographic examination

A standardized periapical radiograph was obtained for each tooth before treatment and during all follow‐up intervals by the main researcher (W.A.) for radiographic examination and evaluation using intraoral x‐ray sensors vatech (EZSensor Classic, Korea).

### 2.3 Sample size calculation

The sample size was calculated using G* Power 3.1.9.4 (Heinrich‐Heine‐Universität, Düsseldorf, Germany) based on the changes in clinical signs and symptoms of the primary necrotic molars. Based on a level of significance of 0.05, a power of 95%, and an effect size of 0.505, a minimum total sample size of 40 patients (20 in each group) was determined to be sufficient, using values given in a previous paper (Nanda et al., 2014).

### 2.4 Randomization

Using a simple randomization method with an allocation ratio of 1:1, necrotic primary molars were assigned to either the conventional root canal treatment group or the 3Mixtatin in NIET group. A random sequence was generated on August 1, 2018, using the website www.random.org.

Opaque and sealed envelopes containing cards with different treatment methods were prepared (20 envelopes per study group), and the children were instructed to randomly select an envelope. This resulted in the assignment of patients to two groups: Group A (control group), which received conventional root canal treatment (*n* = 20), and Group B (study group), which received 3Mixtatin in NIET (*n* = 20).

### 2.5 Blinding

**Figure 1:**
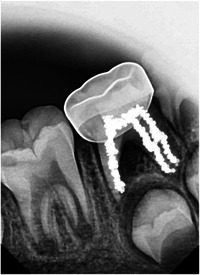
Blinding the treatment method during assessment stage.

Since the current study was an interventional study, the operator could not be blinded to the treatment method used. However, the children participating in the study were fully blinded. Moreover, the treatment evaluation outcomes were conducted by two experienced pediatric dentists (M.L. and Y.A.T.) researchers who were trained and calibrated to the evaluation criteria, and who were also blinded to the type of treatment method used. This was achieved by editing the radiograph images to conceal the root canal, as observed in the Figure 1.

### 2.6 Outcomes measures

The clinical criteria were measured by two external examiners at baseline (T0) directly after the treatment, 1 month (T1), 3 months (T2), 6 months (T3), and 12 months (T4). The criteria included the absence of fistula detected visually, absence of painful symptoms, absence of abnormal mobility evaluated by applying alternate pressure on the outer and inner aspects of the crown of the molar with the aid of two manual instruments, and intact gingival contour detected by palpation.

The radiographic criteria were measured at baseline (T0), 3 months (T1), 6 months (T2), and 12 months after treatment (T3). The criteria included the resolution of furcation lesion radiolucency analyzed on the follow‐up radiographs using ImageJ (Fiji) 2019 software. The absence of pathological bone resorption assessed by detecting any new radiolucency on radiographs, and the absence of pathological root resorption analyzed on the follow‐up radiographs to evaluate the changes in the extent of the radiolucent area.

Two trained pediatric dentists (M.L. and Y.A.T.) evaluated the clinical and radiographic criteria. Before the study, each examiner underwent training and calibration sessions, during which they independently conducted a radiographic assessment of 10 randomly selected non‐vital primary molars twice, with a 2‐week interval, to determine the inter‐ and intra‐reliability of the radiographic evaluations among the examiners. The intra‐examiner and inter‐examiner reliability of the first and second coinvestigators were calculated using Cohen's kappa statistic, which demonstrated a high level of agreement (Table 1).

**Table 1:** Kappa values for intraexaminer and interexaminer reliability in assessing radiographs of non‐vital primary molars.

Reliability type	Examiner	Kappa value
Intra‐examiner reliability	Examiner 1	0.92
	Examiner 2	0.88
Inter‐examiner reliability	Examiner 1 and 2	0.90

**Figure 2:**
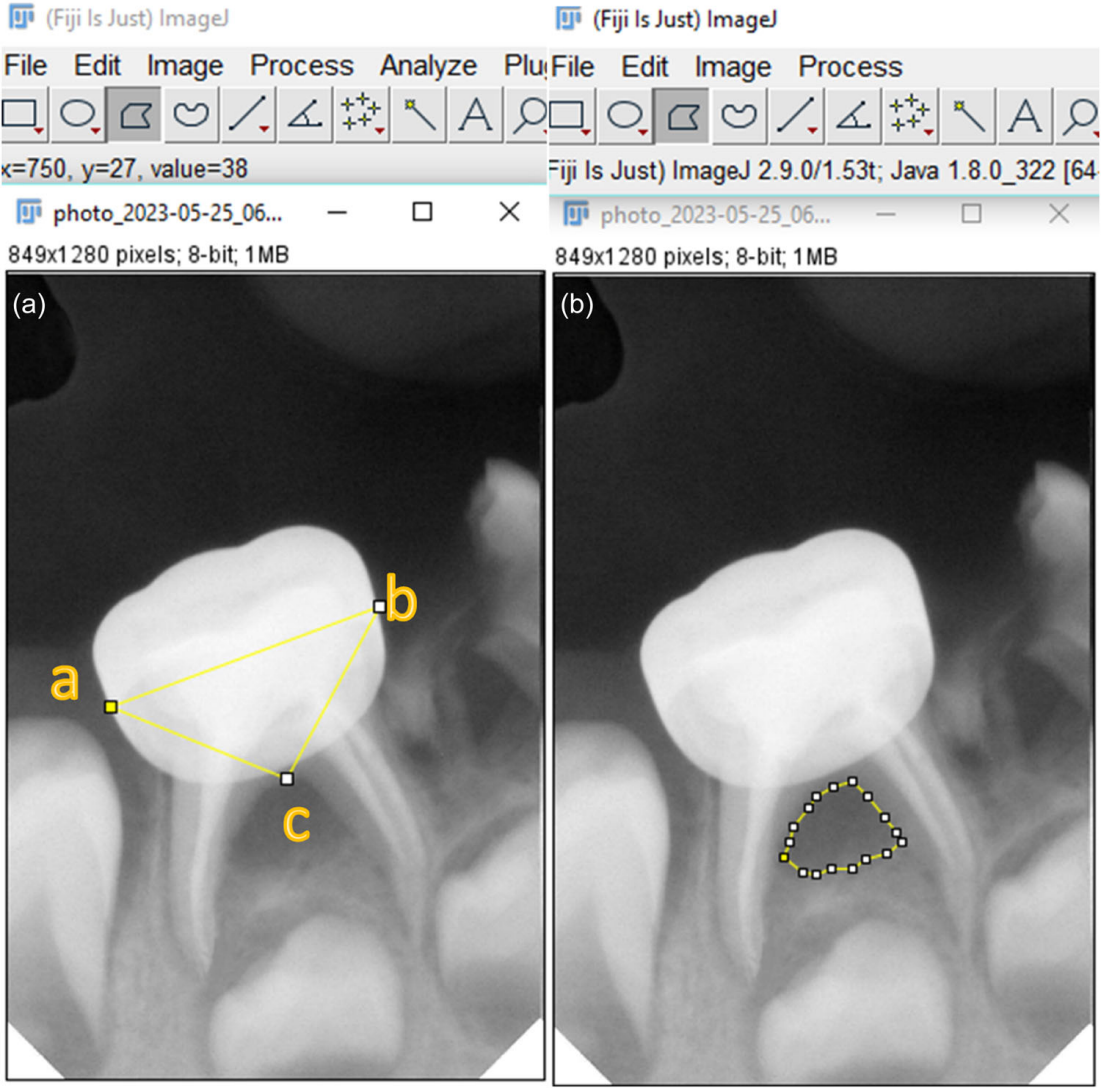
Determining the SSC dimension and the bifurcation lesion size using ImageJ (Fiji) 2019 software; (a) **ab** line: mesiodistal dimension of the crown, **c** point: the furcation point. (b) Calculating the size of bifurcation lesion.

The area of the interradicular radiolucency on the radiograph and the triangle with its base being the mesiodistal dimension of the crown and its apex being the furcation point were calculated. Subsequently, the ratio between these measurements in T0 was determined as a fixed percentage to compare it with the same ratio in T1 and T2 to determine if the lesion size was increased or decreased, no changes, or the lesion was disappeared (Figure 2).

### 2.7 Clinical procedure

Written consent was obtained from all parents of participating children after they were informed about the study's purpose and procedures.

Following the administration of local anesthesia using mepivacaine hydrochloride 3%, each tooth was isolated using a rubber dam. The carious lesions were then removed, and the access cavity was prepared using a bur in a high‐speed handpiece (Bieng. SKU, DGIN21325‐11, China).

In Group A, at the first visit, a conventional pulpectomy was performed using k‐files and h‐files (#15 to #25; Mani, INC. Company) after determining the working length using Woodpex V apex locater. Then, the root canals were treated with 10 mL of 2.5% NaOCl solution, and paper points were used to dry them (Gabadent, Guangdong, China). A Lentulo spiral (Mani, INC. Company) in a low‐speed handpiece was used to place calcium hydroxide dressing into the root canal. A moist cotton pellet was then inserted into the canal to facilitate its setting, and the access cavity was temporarily restored with glass ionomer filling cement (Medifil).

At the second visit after 7 days, after local anesthesia and isolation with rubber dam, the temporary filling, the wet cotton pellet, and calcium hydroxide dressing were removed, then the canals were irrigated with 10 mL NaOCl 2.5%, then with physiological serum and finally with chlorhexidine gluconate 0.2%. Last, canals were obturated with zinc oxide eugenol paste using a Lentulo spiral in a low‐speed handpiece (NSK, SKYSEA, 264378185197).

In Group B, a round bur with a diameter of 1 mm and a depth of 2 mm was used to enlarge the canal orifices. The pulp chamber and the root canal entrances were irrigated with ethylenediaminetetraacetic acid (EDTA) 17% for 60 s to remove the smear layer and increase dentin permeability to chemical solutions and physiological serum. This was followed by irrigation with 10 mL NaOCl 5.25%, then irrigated with normal saline, and finally with chlorhexidine gluconate 0.2%. If any refractory hemorrhage appeared, it was controlled by applying a cotton pellet soaked in NaOCl 10% and maintained for 1 min.

#### 2.7.1 Preparation of modified 3Mixtatin paste

A total of 100 mg of each of three commercially available antibiotics, metronidazole (Metronidazole®, Bahri), cefixime (CEF®, Bahri), and ciprofloxacin (CEPROXENE®, Bahri), were mixed in a ratio of 1:1:1 with 2 mg of simvastatin (SIMVACOR®, Alfares). The coating materials were removed from the drugs, and they were pulverized using porcelain mortars and pestles to obtain a fine powder. Each drug was then stored separately in a tightly capped porcelain container at a temperature of 16°C to prevent exposure to light and moisture for no longer than a week. To prepare the 3Mixtatin, a mixture of one part propylene glycol and seven parts modified 3Mixtatin powder was created to achieve a creamy consistency. This mixture was then applied over the canal orifices and pulpal floor. The preparation of 3Mixtatin was overseen by a qualified pharmacist.

**Figure 3:**
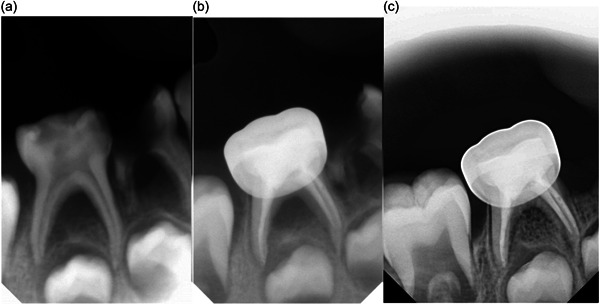
Conventional root canal treatment: (a) before treatment, (b) after 3 months, and (c) after 12 months.

**Figure 4:**
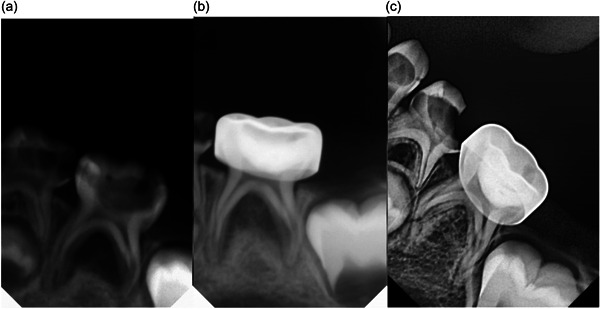
NIET: (a) before treatment, (b) after 3 months, and (c) after 12 months. NIET, non‐instrumentation endodontic treatment.

**Figure 5:**
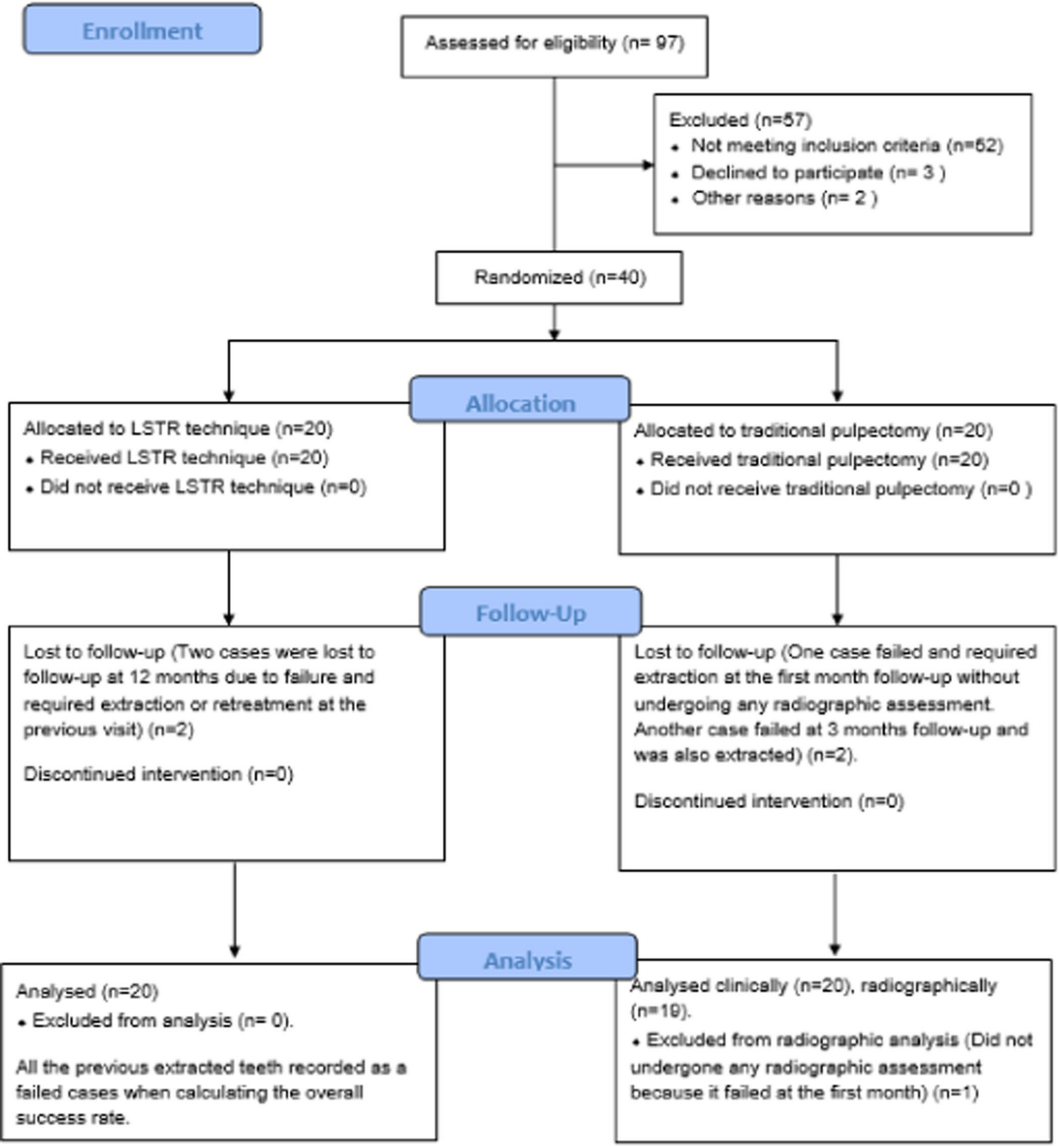
Flow chart of the patients.

After treatment, the teeth in both groups were sealed with a glass ionomer cement and restored with a stainless‐steel crown (3M; ESPE Stainless Steel Primary Molars Crowns). The principal investigator (W.A.) performed all procedures. Clinical evaluations were conducted at 1, 3, 6, and 12 months, while radiographic evaluations were carried out at 3, 6, and 12 months to assess the outcomes of the treatment (Figures 3 and 4). The flow chart of the patient was described in Figure 5.

### 2.8 Statistical analysis

The data collected were organized and analyzed using SPSS software (Version 20, IBM SPSS Inc.). The presence or absence of clinical signs and symptoms between groups was compared using the Chi Square Test, while the Mann–Whitney *U* test was used to compare the groups regarding changes in lesion size (increased, decreased, disappeared, or no changes). The level of significance was set at 0.05 for all analyses.

## 3 RESULTS

Thirty‐eight children, with a mean age of 6.2 (±1.1 standard deviation) years, participated in the study, including 22 males and 16 females.

Before the treatment, there were no statistically significant differences between the two groups concerning the distribution of preoperative clinical signs and symptoms, as well as the size of interradicular radiolucencies on radiographs.

In the first follow‐up visit at 1 month, no clinical signs or symptoms were observed in any of the cases in both groups except one case showed pain on percussion in NIET group that resolve at the next visit and one case failed clinically exhibited abscess, pain and tenderness to percussion in conventional pulpectomy group. Therefore, the total clinical success after 1 month of treatment was 95% for both groups.

Three months postoperatively, 100% of cases showed complete resolution of clinical findings in NIET group versus another case showed acute signs and symptoms (abscess‐spontaneous pain‐abnormal mobility) in conventional pulpectomy group that means the overall clinical success rate at 3‐month follow‐up was 100%, and 90%, respectively, with no statistically significant differences.

At the 6‐month follow‐up evaluation, the first group showed two cases of clinical failure (tenderness to percussion‐abnormal mobility) but in the second group all teeth remained asymptomatic. Which means the overall clinical success rate decreased by 10% (90%) in NIET group.

Both groups exhibited no statistically significant differences in terms of asymptomatic teeth after 12 months of treatment. The total number of clinical failures was 2 out of 20 teeth in each group (Table 2).

**Table 2:** Descriptive statistics of presence or absence the clinical signs and symptoms in the two groups for the two treatment techniques and the *p*‐values of significance testing.

Studied clinical symptom	Studied period	3Mixtatin		Traditional pulpectomy		Chi‐square value	*p*‐value^a^
		Absence	Presence	Absence	Presence		
Spontaneous pain	Before treatment	13	7	15	5	0.476	0.490
	After 1 month	19	1	19	1	0	1.000
	After 3 months	20	0	19	0	–	–
	After 6 months	20	0	18	0	–	–
	After 1 year	18	0	18	0	–	–
Tenderness to percussion	Before Treatment	3	17	1	19	1.111	0.292
	After 1 month	19	1	19	1	1.026	0.311
	After 3 months	20	0	18	1	1.080	0.299
	After 6 months	18	2	18	0	1.900	0.168
	After 1 year	18	0	18	0	–	–
Gingival swelling	Before treatment	11	9	9	11	0.400	0.527
	After 1 month	20	0	19	1	1.026	0.311
	After 3 months	20	0	18	1	1.080	0.299
	After 6 months	20	0	18	0	–	–
	After 1 year	18	0	18	0	–	–
Fistula	Before treatment	13	7	15	5	0.476	0.490
	After 1 month	20	0	20	0	–	–
	After 3 months	20	0	19	0	–	–
	After 6 months	20	0	18	0	–	–
	After 1 year	18	0	18	0	–	–
Abnormal mobility	Before treatment	6	14	2	18	2.500	0.114
	After 1 month	20	0	19	1	1.026	0.311
	After 3 months	20	0	18	1	1.080	0.299
	After 6 months	18	2	18	0	1.900	0.168
	After 1 year	18	0	18	0	–	–

a Chi‐squre test.

### 3.1 Radiographic evaluation

Radiographic evaluations between groups showed no statically significant at 3‐, 6‐, and 12‐month follow‐up (Table 3).

**Table 3:** Descriptive statistics of changing in lesion size of necrotic molars in the two groups for both treatment techniques and the *p*‐values of significance testing.

**Studied period**	**3Mixtatin**					**Traditional pulpectomy**					**Mann–Whitney *U***	***p*‐value** a
	**Lesion size increased**	**No change**	**Lesion size decreased**	**Lesion disappeared**	**Mean rank**	**Lesion size increased**	**No change**	**Lesion size decreased**	**Lesion disappeared**	**Mean rank**		
After 3 months	0	5	5	10	22.50	1	2	14	2	17.37	140.00	.128
After 6 months	2	3	0	15	18083	0	1	3	14	20.25	166.50	.596
After 12 months	0	3	0	15	17.50	0	1	0	17	19.50	144.00	.296

a Mann–Whitney *U* test.

## 4 DISSCUSSION

Successful treatment for necrotic teeth is defined as the disappearance of all clinical signs, along with a reduction in periapical radiolucency and bone regeneration (Duarte et al., 2020). This success of the treatment is influenced by various factors, with the reduction or elimination of bacterial infection being one of the most critical (Moreira et al., 2022).

In this study, it was proposed to use the NIET technique as an alternative to the conventional pulpectomy to shorten the treatment time and maintain children's cooperation. A specific methodology was followed to prove the effectiveness of this technique with the new mixture (3Mix‐Simvastatin) in healing the clinical and radiological symptoms of the necrotic primary mandibular molars. The current study included children aged 4–8 years with adequate root length and less than one‐third physiological resorption, meeting the criteria for conventional pulpectomy. Mandibular primary molars were chosen over maxillary molars to facilitate radiographic assessment, and second molars were preferred over first molars to minimize the anatomical differences.

In the control group of this study, ZOE was chosen as the obturation material based on the latest clinical recommendations by AAPD, which suggested that ZO/iodoform/CH and ZOE may be a superior option for achieving a higher pulpectomy success rate, especially in younger children (Coll et al., 2020). An apex locator was employed to determine the working length with precision and detect any apical resorption. Accurately identifying the actual working length on a radiograph can be challenging due to the anatomical apex being located up to 3 mm from the radiographic apex and frequently occurring on the lateral surface of the root (Winters et al., 2013).

The concept of “lesion sterilization and tissue repair” was originated at the Cariology Research Unit of Niigata University School of Dentistry in Japan (Takushige et al., 2004). A standard mixture of minocycline, ciprofloxacin, and metronidazole has been used in numerous studies and has proven its efficacy (Takushige et al., 2004; Zacharczuk et al., 2019). However, recent studies have replaced minocycline with other antibiotics due to the black discoloration of dentin caused by it (Shetty et al., 2020).

Additionally, other studies have used different mixtures, such as chloramphenicol‐amoxicillin‐zinc oxide powder paste (CTZ). Daher et al. (2015) revealed a low success rate of 27% using CTZ under the LSTR concept. On the other hand, the overall success rate in Moura and colleagues’ (2021) in vivo study applying the same paste was good at 70.5%.

In the current study, cefixime was used as an alternative to minocycline due to its broad‐spectrum antibiotic properties and similar effects. In the practice of this drug mixture, two ratios have been mentioned: 1:1:1 and 1:3:3. In this study, a 1:1:1 ratio was used based on the proportions outlined by Aminabadi and colleagues to prepare 3Mixtatin paste (Aminabadi et al., 2016). However, the effect of this change remains unknown to date (Richa, 2017).

The root canal orifices were enlarged to form medication cavities following Takushige's method (1 mm diameter and 2 mm depth). This was done to ensure proper diffusion of the sterilizing paste to the furcal area and residual necrotic tissue in the root canal system (Takushige et al., 2004). However, other studies have skipped this step and applied the antibiotic paste directly over the pulp chamber floor. According to Parakh study, it is not necessary to remove accessible necrotic pulp tissues in the LSTR technique (Parakh & Shetty, 2019). This is similar to the result obtained by Lokade and colleagues after 12 months of follow‐up, which demonstrate a clinical success rate of 90% without removing the infected accessible necrotic pulp tissue, compared to 90.5% when it was removed. The radiographic success rates were 75% and 76.2%, subsequently, with no statistically significant difference (Lokade et al., 2019).

The modified 3Mix powder was mixed with propylene glycol as a vehicle in a ratio of 1:7 to ensure the best penetration of the paste through dentinal tubules. This was due to the low surface tension property of propylene glycol, which allowed for optimal diffusion of the paste (Cruz et al., 2002).

In the literature, the irrigation protocol varied from no irrigation (Daher et al., 2015) to using copious irrigation with saline (Thakur et al., 2021), NaOCl 2.5% (Zacharczuk et al., 2019), or chlorhexidine 2% (Moura et al., 2021). In the current study, all previous irrigation solutions were used to achieve maximal sterilization of the pulp chamber. Enhancing the permeability of dentinal tubules in the pulp chamber's pulpal floor requires the elimination of the smear layer, as it obstructs the effective penetration of irrigants and medicinal agents (Shetty et al., 2020). This can be achieved by applying an EDTA solution or 35% phosphoric acid, which can enlarge and open up the dentinal tubules (Anila at al., 2020).

In the current study, the clinical success rate for the period of evaluation was recorded at 90% in each group. This result is consistent with the high clinical success rates reported in other studies that have used the LSTR technique. Takushige et al. and Nanda et al. have reported a 100% clinical success rate (Nanda et al., 2014; Takushige et al., 2004). Our results also match those of Lokade and colleagues with 90% clinical success rate (Lokade et al., 2019).

In the study conducted by Jaya and colleagues, abnormal mobility persisted during the first‐month follow‐up visit (Jaya et al., 2012). However, in the current study, all clinical signs and symptoms resolved during the first month in the LSTR group, except for one case that presented with tenderness to percussion, which resolved spontaneously in the next follow‐up visit.

In most in vivo studies, failure is frequently associated with symptom‐free cases (Daher et al., 2015), as observed in this study. Moreover, it was found three asymptomatic cases with static furcation radiolucency in the LSTR group at the end of the follow‐up period, compared to one case in the conventional pulpectomy group. After 12 months of observation, bone regeneration was noticed in 75% of the NIET group and 89.47% of the conventional pulpectomy group. These outcomes differ from those reported by Nanda et al., who found that only 30% of interradicular lesions healed in the standard 3Mix group, compared to 35% in the modified 3Mix group (Nanda et al., 2014).

In the conventional pulpectomy group, early clinical failure was observed at the first‐month follow‐up (one case) and the third‐month follow‐up (one case). This may be attributed to a secondary or persistent infection that caused acute symptoms. In contrast, failure in the LSTR group emerged at 6 months as chronic symptoms, such as tenderness to percussion and abnormal mobility. This may be due to the permanence of an asymptomatic chronic inflammatory condition (Daher et al., 2015), which highlights the role of drugs in suppressing the inflammatory response during the first months after treatment. The late failure may be attributed to the gradual degradation of these drugs in the fluid present in the necrotic pulp, which gives a prolonged antibacterial and anti‐inflammatory effect.

To eliminate any infection, an antibacterial agent with an anti‐inflammatory agent is required. In the current study, the modified 3Mix paste was used as the antibacterial agent, while simvastatin played the role of the anti‐inflammatory agent, as well as a bone regenerative drug. Simvastatin was added to the paste in the current study to potentially improve the radiographic success rate and promote inter‐radicular lesion healing (Aminabadi et al., 2016; Thakur et al., 2021). In dentistry, simvastatin has regenerative effects and improves bone formation by activating osteoblasts while suppressing the function of osteoclasts, resulting in a healing process (Soares et al., 2018).

Thakur and colleagues (2021) conducted a study similar to this one, in which they evaluated the efficacy of a similar mixture to treat chronic necrotic primary molars and compared it with modified mixture of (cefix‐ornidazole‐ciprofloxacin) and Metapex pulpectomy. At the last follow‐up after 1 year, the pulpectomy group showed the greatest number of failures, with a success rate of 42.9%. However, LSTR had the highest success rate with 90.6%, followed by 60.9% for both 3Mixtatin and modified 3Mix‐MP, respectively. When compared to this study, pulpectomy with ZOE has proved its efficacy with an overall success rate of 85% at the end of the follow‐up period, versus 3Mixtatin, which had a lower success rate of 75% at the 12‐month interval, but this was not statistically significant. Thakur and colleagues (2021) explained the decreased success rate with Metapex pulpectomy by stating that the use of calcium hydroxide in poor prognosis molars enhanced the inflammatory response, leading to increased root resorption.

The main limitation of this study was the limited duration of our follow‐up period, we were unable to investigate the prolonged effects of NIETs on accelerated root resorption, early exfoliation of deciduous teeth, and their potential impact on the eruption of permanent successors. The current results need to be supported by further histological studies to determine the nature of the bone‐restoring lesion.

## 5 CONCLUSION

Within the above‐mentioned limitations of this study, it was determined that NIET technique using 3Mixtatin presents a promising alternative to conventional pulpectomy in treating necrotic primary molars. It is crucial to emphasize that the decision to use LSTR in necrotic primary molars should be made after careful evaluation by a dental professional, taking into account factors such as the extent of infection, the condition of the tooth, and the overall oral health of the patient. Finally, we recommend the protocol used in this study for the LSTR technique.

## AUTHOR CONTRIBUTIONS

Walaa Almarji conceptualized the idea, provided the treatment, and contributed to the writing and documenting. Mohannad Laflouf conceptualized the idea and supervised the MSc thesis for Walaa Almarji. Yasser Alsayed Tolibah contributed to the interpretation of data and the revision, formatting, and re‐editing of the manuscript. All authors read and agreed to the published version of the manuscript.

## CONFLICT OF INTEREST STATEMENT

The authors declare no conflicts of interest.

## Data Availability

Data can be requested by an email to the corresponding author.
